# Acknowledgment of Reviewers

**DOI:** 10.14252/foodsafetyfscj.D-24-00017

**Published:** 2024-12-20

The Editor-in-chief of ***Food Safety*** expresses his gratitude to the following experts for their invaluable assistance with review of manuscripts in **Vol. 10 to 12**.

**Table:** 

AKAMATSU Rie	KAWANISHI Toru	NOHMI Takehiko	UNEYAMA Chikako
AKIBA Masato	KIMURA Bon	ONO Michiyuki	YAENO Takashi
HACHINOHE Mayumi	KONDO Kazunari	ONO Ryuichi	YAMADA Takashi
HIMENO Seiichiro	KUMAGAI Susumu	SATOH Hiroshi	YAMAMOTO Shigeki
ISHIDA Seiichi	KUWAGATA Makiko	SHIBATA Norihito	YAMAZOE Yasushi
ISHIHARA Kanako	MATSUNAGA Tamihide	SOBUE Tomotaka	YOGO Yasuhiro
ITO Kiyomi	MURATA Masatsune	TABEI Yutaka	YOSHIKAWA Nobuyuki
KAWAMOTO Shinichi	NABESHI Hiromi	TERAJIMA Jun	YOSHINARI Kouichi
KAWANISHI Michiko	NAKAJIMA Miki	TOHKIN Masahiro	

